# A time-varying biased random walk approach to human growth

**DOI:** 10.1038/s41598-017-07725-4

**Published:** 2017-08-10

**Authors:** Béla Suki, Urs Frey

**Affiliations:** 1 Department of Biomedical Engineering, Boston University, 44 Cummington Str., Boston, Massachusetts 02215 USA; 2 University Children’s Hospital Basel, UKBB, University of Basel, Spitalstrasse, PO Box 4031 Basel, Switzerland

## Abstract

Growth and development are dominated by gene-environment interactions. Many approaches have been proposed to model growth, but most are either descriptive or describe population level phenomena. We present a random walk-based growth model capable of predicting individual height, in which the growth increments are taken from time varying distributions mimicking the bursting behaviour of observed saltatory growth. We derive analytic equations and also develop a computational model of such growth that takes into account gene-environment interactions. Using an independent prospective birth cohort study of 190 infants, we predict height at 6 years of age. In a subset of 27 subjects, we adaptively train the model to account for growth between birth and 1 year of age using a Bayesian approach. The 5-year predicted heights compare well with actual data (measured height = 0.838*predicted height + 18.3; R^2^ = 0.51) with an average error of 3.3%. In one patient, we also exemplify how our growth prediction model can be used for the early detection of growth deficiency and the evaluation of the effectiveness of growth hormone therapy.

## Introduction

Growth and development are complex nonlinear biological processes^1^. The growth process is driven by gene-environment interactions allowing for heterogeneity on a population level as well as adaptability to environmental conditions^2, 3^. At the level of individual organisms, defects in growth can lead to the emergence of different health and disease phenotypes^4–6^.

Various models have been proposed to describe growth as a function of time including exponential, sigmoid or even more complex mathematical functions most of which are descriptive in nature^7–10^. A universal mechanistic model was developed by West *et al*.^11^ in which growth is related to total metabolic rate partitioned for cellular maintenance and cellular growth. This theory predicts an exponential growth for the quarter power of body mass. The key assumptions of the West theory^11^ are based on the concept of continuous growth and allometric scaling but it does not fully explain human^12^ and invertebrate^13^ growth nor does it account for individual variability and adaptability. To better understand variations within a population as well as adaptability of the individual, it is necessary to relate mechanisms influencing growth operating on short time scales at the microscopic level to the overall growth of an organism and its adaptations to environmental fluctuations during development.

Observations of growth pattern in human infants provided evidence that growth is not continuous, but it occurs in saltatory bursts separated by waiting periods of nearly no growth^14^. The temporal dynamics of growth pattern suggested a non-random aperiodic behaviour^15^ and mechanistically, this pattern^16–19^ may be related to the pulsatile nature of growth hormone expression^20–22^ or cell cycle dynamics of mitotic start and stop phases^23^. Although continuous and salutatory growth may co-exist but because of the limited accuracy of measurements, it is difficult to resolve whether the two growth patterns are clearly distinct^15, 24–26^. Nevertheless, there is evidence that salutatory growth exists in infancy^14^ and even in school age^24^ although the exact distributions of salutatory intervals and growth amplitudes are still largely unknown.

Another general limitation of most growth models is that they are unable to fit population mean and variability curves^8, 19, 27^, or the heterogeneous behaviour of growth trajectories of individual subjects^15, 26^. Furthermore, there is also no clear association between the variability of individual growth and the variability of population growth, and thus population based growth charts are not suitable to predict the variability of individual growth trajectories. Nevertheless, the latter issue may be of critical importance in clinical decision making^28^. In clinical practice, individual growth trajectories, including confidence boundaries, would be needed to detect early signs of abnormal growth due to chronic disease, or to judge the efficiency of treatment strategies such as growth hormone therapy. For clinical decisions in the context of precision medicine, we need individual growth prediction models including confidence boundaries rather than population based growth estimates.

In this study, we aim to link micro-scale events to overall growth pattern at the individual as well as population levels during preschool childhood development. Often referred to as a ‘window of opportunity’, preschool age is a critical developmental period^5, 6, 29^, during which complex chronic diseases can arise due to interaction of genetic and environmental factors often accompanied by growth disturbance. To this end, we develop a biased random walk model of individual growth in which the growth increments are taken from time varying distributions mimicking the bursting behaviour of observed saltatory growth patterns. The model is then extended to include genetic factors as well as the effects of possible variations in environmental conditions both at the individual and population levels. We train the model based on existing World Health Organization (WHO) growth charts^27^, and test its predictive ability by computing 5-year growth trajectories of individual subjects in an independent prospectively assessed longitudinal dataset from our laboratory. Finally, we exemplify the clinical utility of the modelling approach in a prematurely born small child with growth deficiency.

## Results

**Figure 1:**
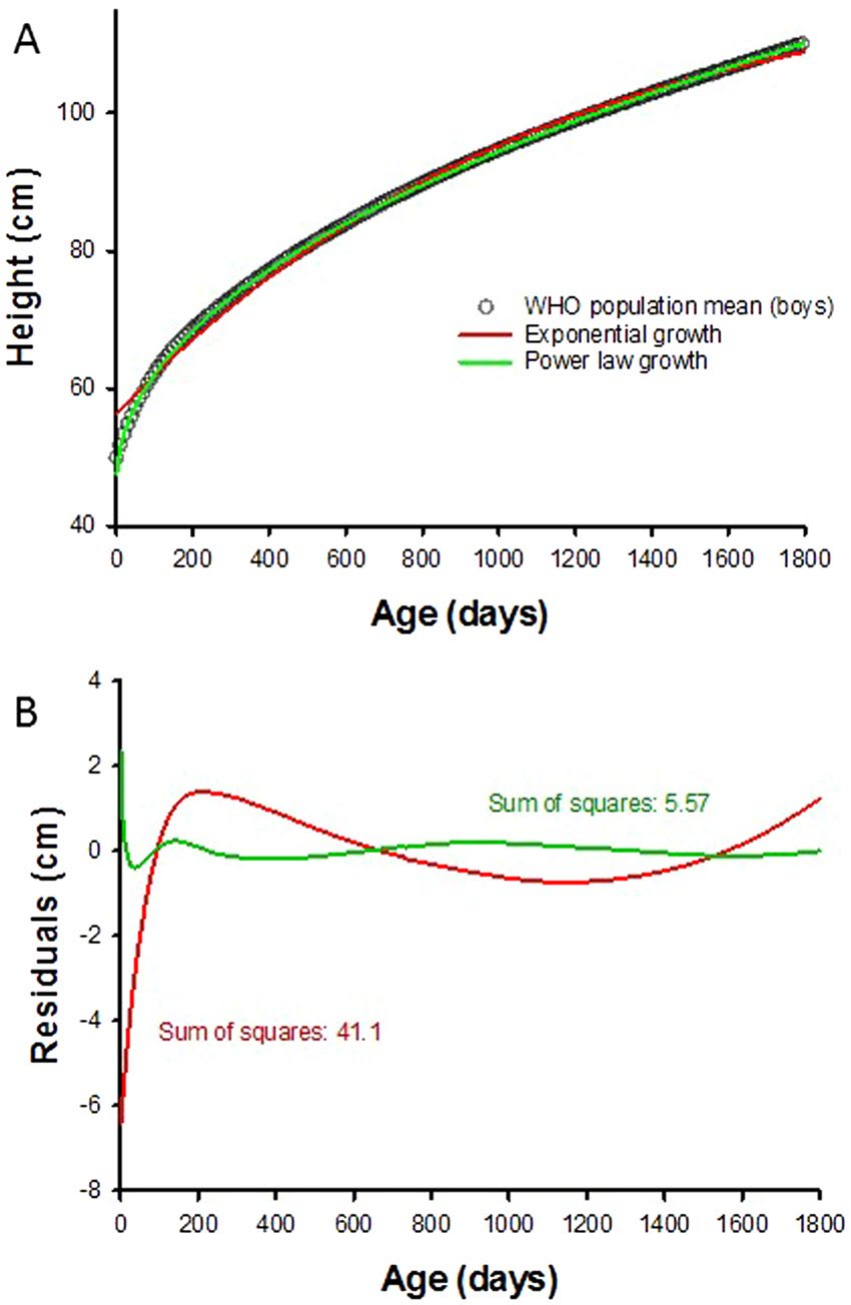
(**A**) Comparison of the fits of the exponential (red) and power law (green) growth models to the population mean of boy’s height in the World Health Organization (WHO) data set. (**B**) Comparison of the sum of square residuals of the exponential and power law model fits. Note that the error of the exponential model is more than 7 times larger than that of the power law model.

We developed a stochastic model of biological growth based on the idea that growth occurs in discrete steps and can thus be described as a random walk. The steps of the random walk are taken from a distribution with time varying parameters with a positive mean. The model also includes a feedback system which determines when the distribution of step sizes changes. Specifically, a time window (w) is defined during which the parameters of the distribution do not change. The random walk starts with initializing the mean (μ) and variance (σ^2^) of the distribution and generates w number of steps. Since the growth must approach a finite value, μ takes a smaller value after w steps gradually approaching 0. The cumulative running sum, which is also the position of the random walker, represents the growth curve. The model is solved analytically for two specific cases that describe how μ decreases in time: an exponential and a power law decrease (see Methods). Both models have 3 parameters that were adjusted to fit the mean growth curves of the WHO data set. The power law model provides a significantly better fit with a residual sum of squares 7 times smaller than the exponential model (Fig. 1). Thus, for the analysis of individual subject’s growth curve, we only retain the power law model and extend it computationally as follows.

**Figure 2:**
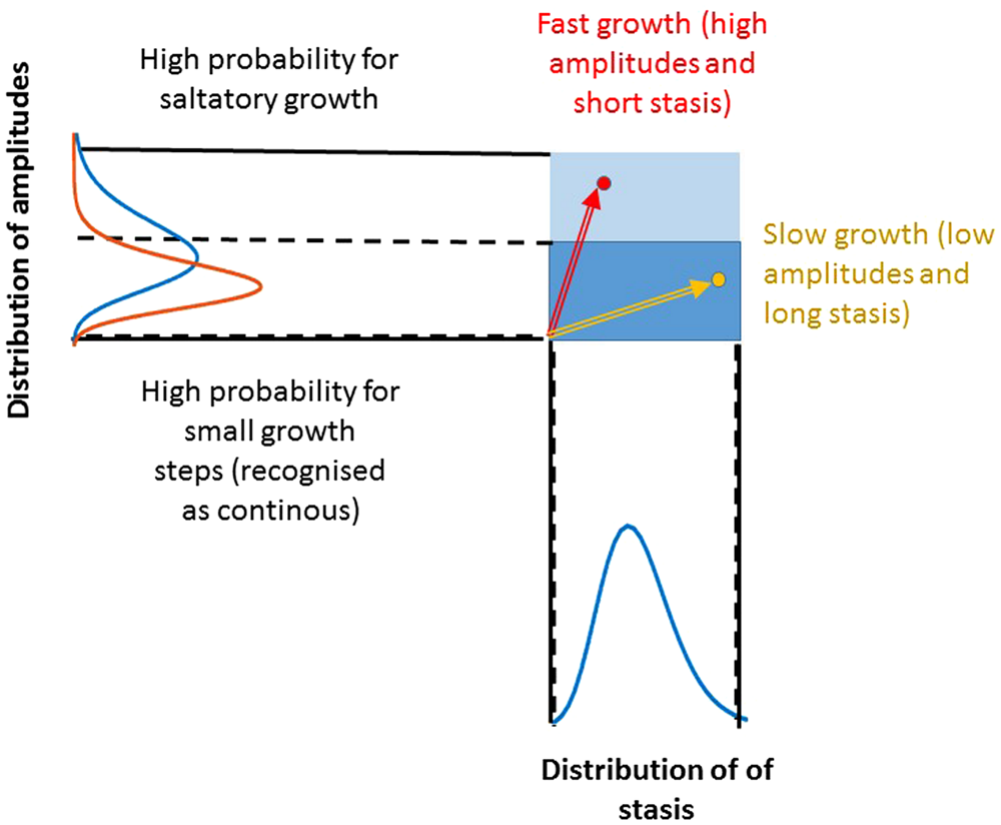
Schematic of the growth process modelled by the time-varying biased random walk. The distribution of stasis times is shown on the x axis by the blue lognormal curve. Two distributions of growth increment amplitudes are shown by the lognormal curves on the y axis. The blue represents a faster growth with larger increments at an younger age while the brown demonstrates a later stage of growth with smaller increments. For the stasis time, only one curve is shown as experiments do not suggest that this distribution changes with age. On this diagram, the red vector represents fast growth with short stasis time and large increments whereas the yellow vector corresponds to slow growth.

The computational model incorporates numerically a growth rate function that determines the time varying bias. Since the growth curve follows a power law with an exponent 1 + α (see Eq. 10, Methods), the growth increment, which is the derivative of the growth curve, is also a power law with an exponent α. We therefore prescribe that the growth increments are taken from a lognormal distribution with a mean that decreases with time after w steps according to a power law with an exponent α which is obtained by fitting the derivative of the mean growth curve of the WHO data set. The variance of the lognormal (σ^2^_ampl_) is obtained from fitting (see below). Additionally, we introduce stochastic fluctuations in the time intervals between growth steps, the stasis times, which are also modelled as a lognormal with a given mean stasis time (T_stasis_) and variance (σ^2^_stasis_). Figure 2 illustrates how these distributions determine the overall growth rate.

**Figure 3:**
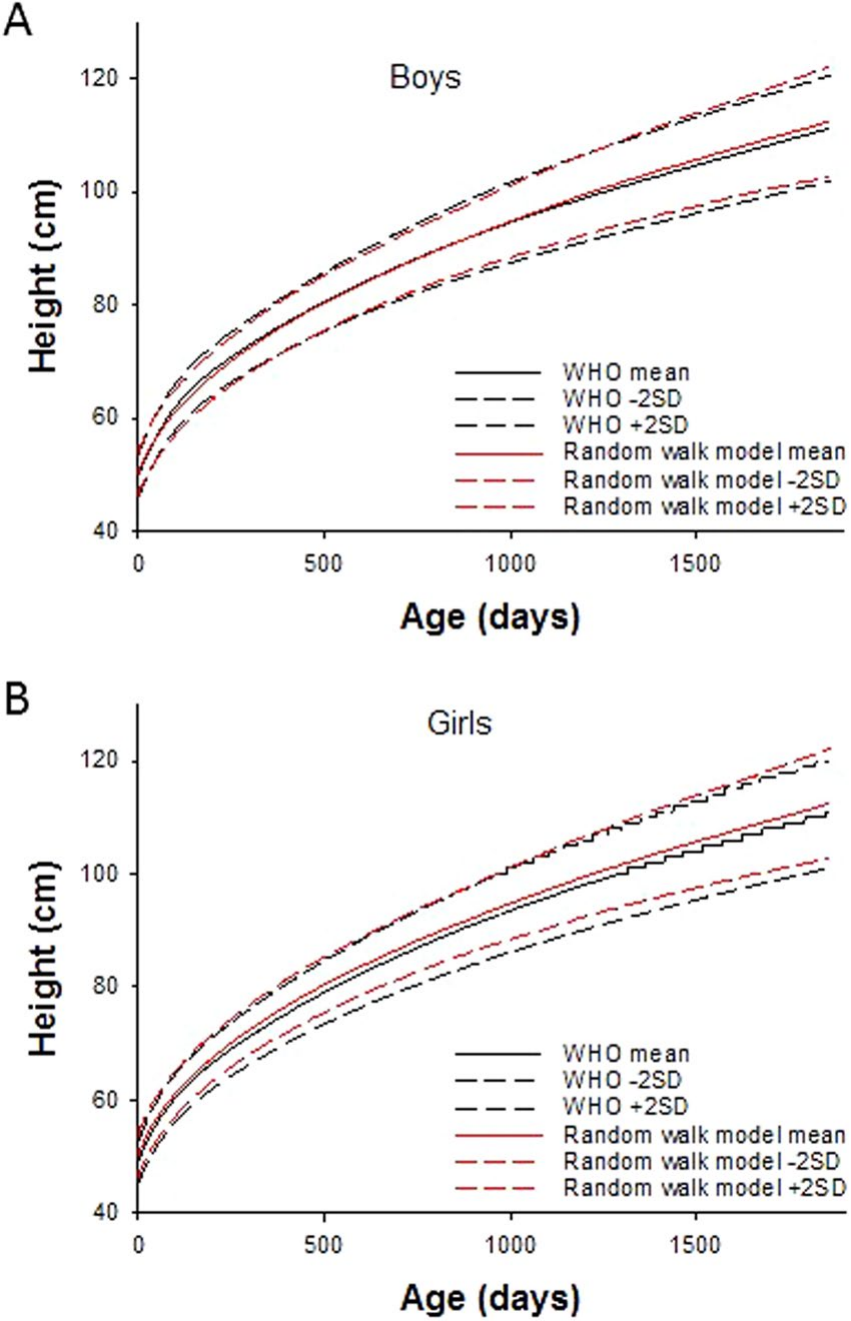
Simulated means and 2SDs of growth curves in comparison with the WHO training data set for boys (**A**) and girls (**B**).

Using a grid search, we fit the mean as well as the mean ± 2SD height curves in the WHO data set with our computational model. Accordingly, we find that for boys, the optimal values for T_stasis_, σ_stasis_ and σ_ampl_ are 10 days, 0.1 days and 0.3 cm, respectively, whereas these numbers for girls are 11 days, 0.2 days, and 0.3 cm, respectively. While the model fit the mean height curve of the WHO data well, the 2SD curves were best fit if we also allowed an additional random variation in the genetic component. To do this, the value of α was randomized within ±2.5% of the value that corresponds to the height at birth for each individual run for a given subject. With these parameters, the overall error of the fit for population mean was 0.69 cm and for the ±2SD it was 0.99 cm. Figure 3 compares the simulated mean and ±2SD height growth curves with those in the WHO data set in boys (A) and girls (B).

**Figure 4:**
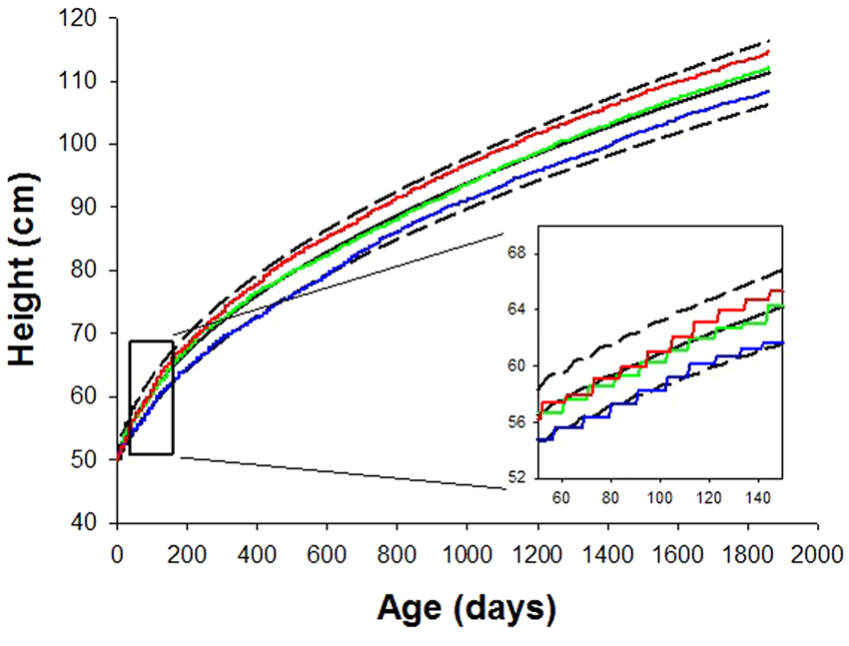
Simulation of a family of individual random walks (red, green and blue lines) of a given subject starting from birth. The probabilistic simulation allows us to calculate the mean (black solid line) as well as confidence intervals (+SD, black dashed line). The inset shows a magnified region of the growth with the 3 discrete random walk realizations. Note the staircase-like growth curves corresponding to the bursting behaviour.

**Figure 5:**
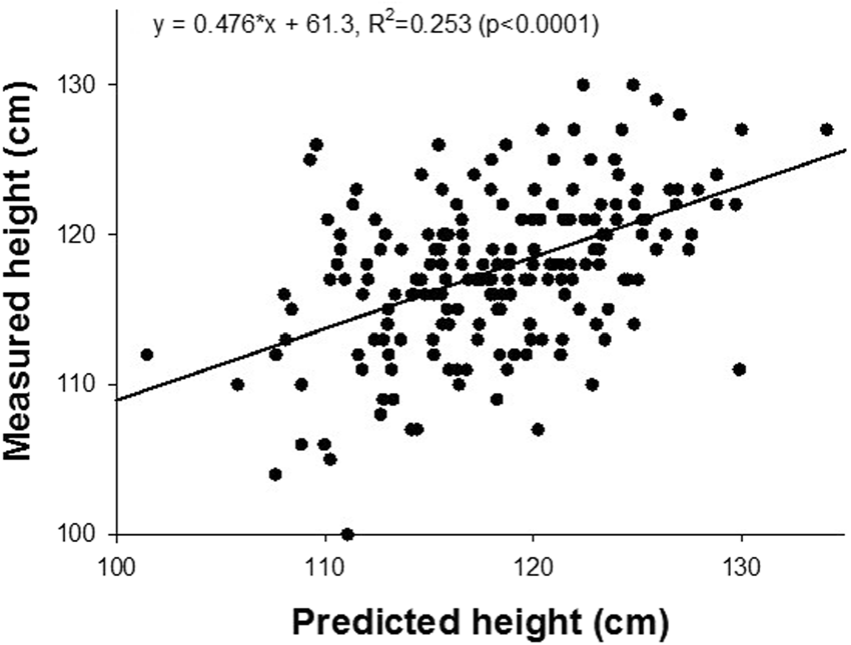
Measured height at 6 years of age as a function of predicted height computed from height at age of 1 month in 190 children from the BILD data set.

For clinical decision making, individual growth trajectories with confidence intervals are needed. Our fully established computational time-varying biased random walk model allows us to simulate a family of random walks for a subject starting from any age if the value of α is known. To obtain α, we first carry out a regression between the α values obtained from the fitting of Eq. 10 to the WHO mean, the mean ±SD and the mean ± 2SD curves and height at 1 month of age. For any given child, this approach provides individual runs as well as an estimated mean trajectory and SD curves (Fig. 4). These trajectories are based on the assumption that the individual child statistically behaves similarly to the reference population in the training set. It can be seen that the bounds around the mean increase as a function of time. The individual trajectory is based on the height at the chosen initial age and the allowed variations correspond to the population-based variations derived from the WHO data. We next launch 500 trajectories using the height at 5 weeks of age in 190 subjects from the BILD (Bern Infant Lung Development) data set (Table 1)^29^ and compare the predicted and measured heights at 72 months of age (Fig. 5). While there is no difference between the predicted and measured heights with an average error of 3.9%, the regression slope is 0.476 (p < 0.0001), the R^2^ is only 0.253.

**Table 1:** Biometric data of the BILD cohort.

	Mean	Median	SD	CI
N	190			
sex (boys/girls)	107	56.3%		
Gestational age (weeks)	39.82	40.00	1.19	0.03
Height at birth (cm)	49.57	50.00	2.06	0.04
Age at first measurement (days)	35.40	35.00	5.27	0.15
Height at first measurement (cm)	55.05	55.00	2.38	0.04
Z-Scores (SDS, WHO)	0.07	0.10	1.09	14.50
Age at second measurement (years)	6.02	6.05	0.33	0.05
Height at second measurement (cm)	117.65	118.00	5.56	0.05
Z-Scores (SDS, WHO)	0.39	0.43	1.03	2.62
Predicted height at second measurement (cm)	117.09	117.25	5.48	0.05
2 standard deviation of prediction (cm)	2.64	2.62	0.26	0.10

Height at 6 years was predicted from length at 5 weeks of age (n = 190). SD: standard deviation, CI: confidence intervals, Z-Scores SDS: standard deviation scores from age related height based on the WHO reference values^27^.

To improve on the prediction, we note that there are environmental influences that can also change both the initial and later growth rates. In order to allow adaption to such fluctuations, we build an adaptive correction mechanism into the model using serial observations. The difference between model prediction and measured height at any given age is likely a consequence of external influences such as feeding pattern or diseases. We account for such effects by adaptively correcting the value of α as follows. We first carry out a regression between the α values obtained from the fitting of Eq. 10 to the WHO mean, the mean ± SD and the mean ± 2SD curves and height at 1, 2, 4, 6 and 12 months of age. The procedure is the same as that described above for the initial birth height. This allows us to set up a look-up table of population α and intercept values at these time points. Next, given the height and the corresponding α at t_1_ (e.g. 1 month of age), we use our model to predict the full distribution of height at the next measured age t_2_ (e.g. 2 months). Comparing the measured height to the predicted distribution of height, we determine the Bayesian probability p_h_ that the measured height at t_2_ is the actually observed value. This allows us to adjust the model and launch a set of new trajectories starting from t_2_ as follows: (1) α is corrected by a small amount depending on the distance between the previous value, α(t_1_), and the current value from the look-up table, α(t_2_), as follows:

$${\rm{\alpha }}^{\prime} ({{\rm{t}}}_{2})={\rm{\alpha }}({{\rm{t}}}_{1})+{\rm{c}}[{\rm{\alpha }}({{\rm{t}}}_{2})-{\rm{\alpha }}({{\rm{t}}}_{1})](1-{{\rm{p}}}_{{\rm{h}}})$$

and (2) we start the new trajectories from the measured height at t_2_ using the corrected α’. The value of c = 0.05 was found by comparing predictions and measured height. Note that if the predicted and measured heights are very close, p_h_ is nearly 1 and no correction is required. With any subsequent observation, we thus reset and narrow the probability distribution of observing future height values. Also notice that the entire history of measured height values influences the adaptive predictor through the above equation.

**Figure 6:**
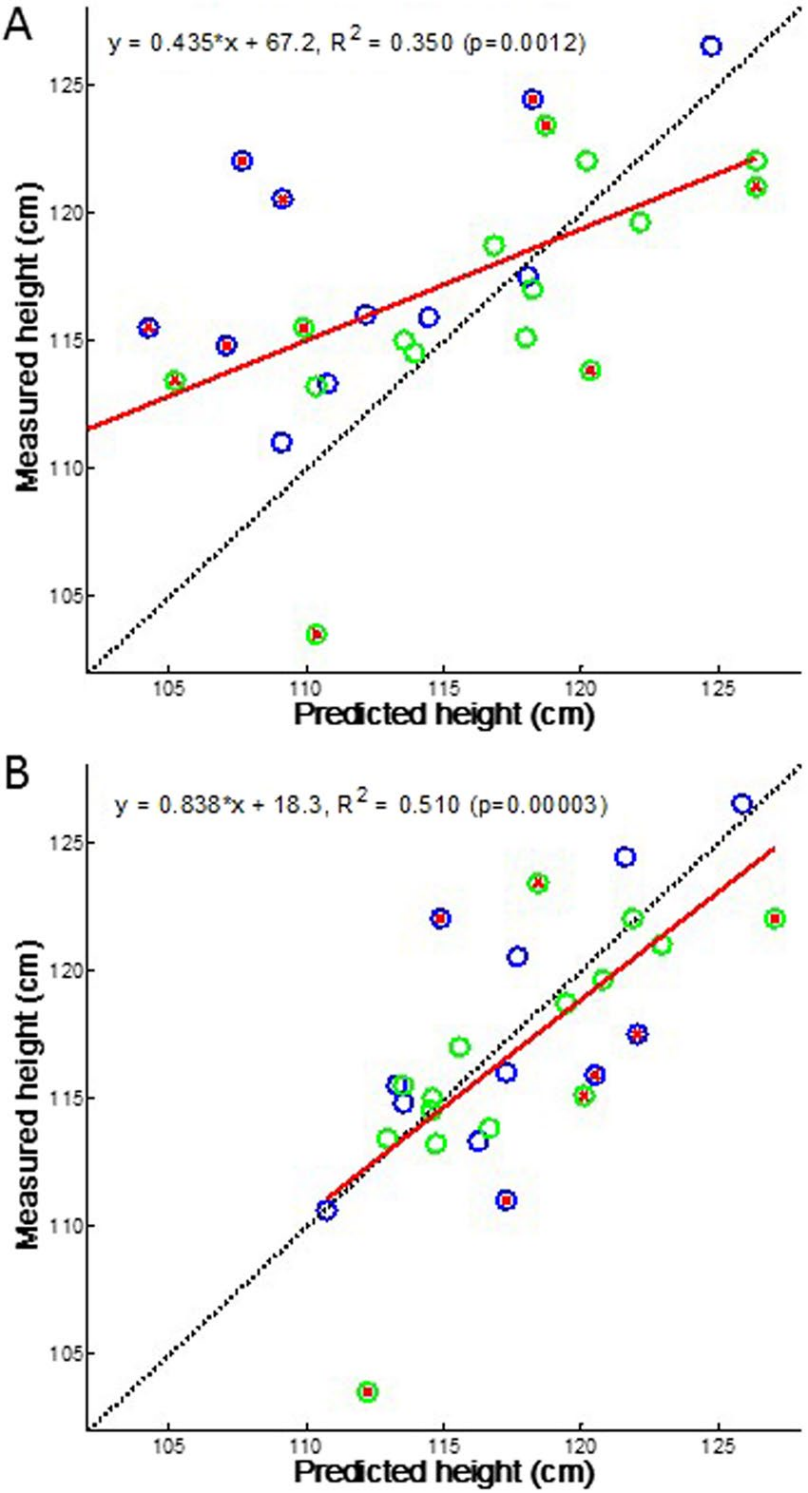
Prediction of measured height in the subset of 27 subjects who had complete data set between birth and 12 months of age. (**A**) Prediction of height at 72 months of age with the time-varying biased random walk model using 500 realizations and utilizing height only at 1 month. (**B**) Adaptive prediction of height at 72 months of age with the time-varying biased random walk model using 500 realizations as well as utilizing the growth history including height values at 1, 2, 4, 6 and 12 months of age in the same 27 subjects. Note that the adaptive Bayesian approach significantly improves the overall long term predictability of the target height by including a history of previous observation. Dotted line: line of identity; red line: regression line; blue circles: boys’ height; green circle: girls’ height; red cross in circles: those subjects whose measured height was outside the mean ± SD of the prediction.

To test the predictive ability of the model on an individual basis, we calculate the individual growth trajectories and the corresponding ±SD curves for a subset of 27 subjects of the BILD data set for which multiple measurements were available (Table 2). These simulations are based on 500 realizations of the random walk model. Figure 6A demonstrates the predictive ability of the model when the growth history is not taken into account. The slope of the regression line is somewhat reduced compared to the full data set but the R^2^ increases to 0.35 (p = 0.0012). Next, we utilize the history of growth below one year to predict height at 6 years using our adaptive correction method applied at 1, 2, 4, 6 and 12 months of age (Fig. 6B). In this case, substantially more of the 27 children are found to grow within their individual bounds of growth trajectory. Furthermore, the regression line has a slope much closer to unity [measured height = 0.838*predicted height + 18.3] with a significantly increased R^2^ = 0.51 (p < 0.00003). Additionally, paired t-test shows no difference between measured and predicted heights with an average error of only 3.3%.

**Table 2:** Height data of a subset of the BILD cohort and model predictions.

	Mean	Median	SD	CI
N	27			
sex (boys/girls)	12	44.4%		
Gestational age (weeks)	39.9	40	1.05	0.41
Height at birth (cm)	49.8	49.5	1.78	0.71
Height at 1 month (cm	53.2	53	2.4	0.95
Height at 12 months (cm)	74.5	73.5	2.48	0.98
Height at 6 years (cm)	116.9	115.9	4.94	1.95
Model predicted height at 6 years (cm)	117.6	117.3	4.21	1.67

Definitions are given in Table 1. There is no statistically significant difference between measured and model predicted height at 6 years (paired t-test).

**Figure 7:**
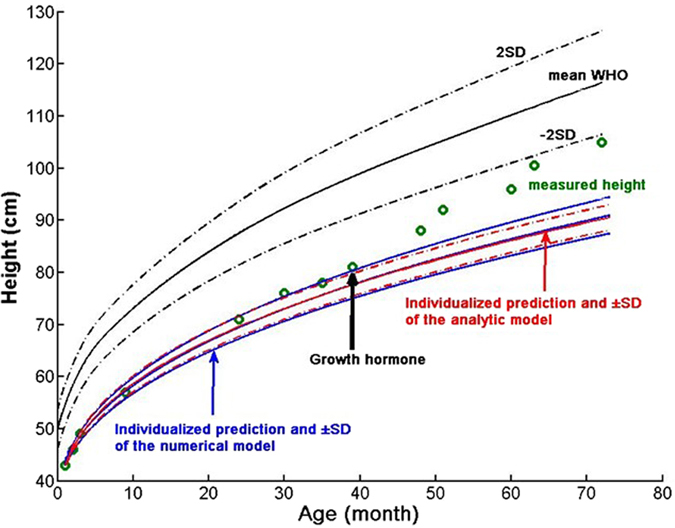
Measured (green circles) and predicted height (blue solid line) and ±SD (blue dashed lines) in a girl born prematurely and small for gestation, following the upper limit of her personal growth trajectory and rapidly exceeding her predicted trajectory after starting growth hormone therapy at the age of 4 years. The WHO mean and ±2SD curves for girls are also plotted (black). For comparison, the predictions of the analytical model are also shown (red).

Finally, we demonstrate how our model can be used to detect growth deficiency. Figure 7 compares the time course of measured heights with the predicted trajectory in a female subject born prematurely and small for gestation. The model captures well the measured time course until 39 months of age when the subject started receiving growth hormone therapy. First, when compared to the WHO mean-2SD, it is apparent that the model predicts that the subject would not reach normal height much earlier than she actually was given therapy. Second, the model can be used to detect the efficiency of the therapy as the model can predict the trajectory without therapy. Lastly, for comparison, the much simpler analytical model-based prediction of height and SD are also plotted demonstrating a good agreement with the full numerical model.

## Discussion

We have developed a probabilistic, adaptive growth model based on a time-varying biased random walk concept. The model was trained on the WHO population height curves. We also derived an analytical solution for the mean and SD of the growth model. Model parameters were estimated by fitting the WHO height curves including their population percentiles. To our knowledge, this is the first model to relate the discrete salutatory nature of individual growth trajectories to the variability of population growth curves. Using the model, we are also able to construct a family of potential growth trajectories for an individual child and hence we can estimate the entire probability distribution of predicted height and hence derive confidence intervals that a predicted target height will be reached within a given time period. In an independent dataset of 190 Swiss children from our birth cohort^29^, we compared the predicted target heights at 6 years from measured data at 5 weeks and compared them to the prospectively measured heights at 6 years. We found a good correlation between prediction and measured outcome with an average error of only 3.9% and in a subset of 27 subjects the error dropped to 3.3% when an adative approach was applied.

### Clinical implications

As an example of the power of modelling approach, we analysed the growth of a single child born prematurely and small for gestation. Her natural growth followed the upper limit of the trajectory predicted by our model. It is clinically important that the predicted trajectory did not reach the WHO population’s 3^rd^ percentile. This implies that the child had limited natural chances to reach normal height during growth. The decision to use growth hormone therapy could have been made earlier had such a prediction been available for the clinician. Additionally, after the start of growth hormone therapy, the measured height immediately exceeded the upper bound of the model predicted height. Given that growth hormone is not always effective in infants born prematurely and small for gestation, obtaining immediate evidence of treatment response is critical since potential side effects of treatment and costs need to be considered.

Classical growth models are based on normative age related height or weight distributions and the prediction of individual growth is always related to the population’s age related height distribution. Population based growth charts are not able to account for the large variability of individual growth patterns^26^. Although regression based models are available^7, 9, 10, 30^, to our knowledge, no adaptive mechanistically based prediction model exists which could predict an individual’s growth from any given starting age and which also provides individualized probability that the target height will be reached. In addition to growth hormone therapy, the latter is particularly important in other clinical decision making processes. For example, in paediatric pharmacology^28^, a long standing problem is that the general practitioner needs to dose medication without the knowledge of current height or weight, but with biometric measurements made at the last visit in the general paediatric practice. To estimate the risk that the dosage is incorrect, it is necessary to know the probability that the prediction of the current height or weight is true. If the probability of a correct prediction is low, the patient’s actual height or weight needs to be assessed before medication. Similarly, in situations of treating sick children with growth deficits (Fig. 7), probabilistic estimates of the time interval beyond which a significant improvement in the child’s growth pattern is expected helps medication planning. Furthermore, environmental conditions (e.g. nutrition, toxic substances) may change during a child’s development. In such cases, the estimated growth cannot not easily be derived from WHO growth data^27^. In current clinical practice, the prediction of target-height is only based on paternal height measures and end-height predictions are based on bone X-rays. Both methods, however, do target height in adolescence. For early preschool age, however, our adaptive, probabilistic growth prediction may be more useful and can adjust for environmental influences and take into account the information content of the history of serial past height measurements.

### Limitations of the methods and the model

The concept of time-varying biased random walk is useful and can be extended to predict other growth curves. For simplicity, we have restricted the model to height prediction within the preschool age range although prediction of weight gain is equally important. Future studies should address the issue of the more complex weight gain characteristics in this age range. Nevertheless, the concept could be adapted to older age ranges where hormonal factors and puberty significantly complicate the prediction. Such predictions may require more complex time-varying increment distributions than the power law applied here. While there are proposed mechanisms for the salutatory growth such as pulsatile growth hormone expression^20–22^ and cell cycle dynamics^23^, our model only incorporate the discontinuous nature of growth at the level of the organism.

We have also made several assumptions, which need to be considered in future work generalising the method for other age ranges. At birth, length within the WHO data set is assumed to be normally distributed. We also assumed that the birth height is determined genetically although feeding and environmental fluctuations probably contribute significantly. To account for the population variability, it was necessary to introduce a small intra-individual random variability (2.5%) of growth rate for each subject. Furthermore, most parameters of the model were derived from fitting the training set of the WHO data. Although this is a limitation of the approach, the WHO data set has been shown to be appropriate for a wide range of situations^27^. If data specific to the country or region were available, these parameters may be fine-tuned for a given type of population in future studies. This limitation together with the unknown environmental factors such as feeding pattern may explain the fact that the model is able to capture only about half of the variance of the individual data (Fig. 6B). Additionally, the biological evidence and quantitative description of saltatory growth and waiting time distributions is limited especially in human subjects. The best evidence is in infancy and early childhood^14, 17, 24^ but data for older age groups to allow quantification of the width of the random walk distribution are scarce. Any new data, however, can be easily incorporated into the framework of the current model by changing the distributions or improving on the adaptive correction. For example, in case the growth is more continuous in a certain age, the distribution of increments would need to peak around a small value and the stasis times should be in the same order of magnitude as the time resolution of the measurements. In this case, the computational model simply reduces to a continuous growth model without discrete steps. The analytical model, however, has a general form that does not depend on the knowledge of the exact distributions of the salutatory steps (Eq. 10). If more specific information on salutatory and continuous growth patterns become available, future studies could improve the predictive power of our computational model. The use of lognormal distribution of waiting times may be related to how random walks with a drift lead to lognormal escape times^31^. Finally, the calculation of the SD in the analytical model assumes that the increments are uncorrelated. In its current form, the analytical model can only be used to predict the SD and hence it is not possible to apply in an adaptive manner which is why it was only used for the individual child’s case in Fig. 7.

In summary, our probabilistic, adaptive growth model represents a novel approach to understand and quantitatively predict growth and development in young children. The model links discrete growth in individuals to continuous population growth curves and is of high relevance for clinical decisions in the context of precision medicine. To our knowledge, this is the first individualized growth prediction algorithm, which can be performed based on previous history of observations. This improves the safety of growth predictions for clinical decision making such as drug dosing. Furthermore, the statistical predictions by the model could be automated for tele-monitoring purposes combined with automatic learning from each observed height. Such an approach may also help detect and better quantify the probability of upcoming growth deficits in chronic diseases at an early stage and the effects of subsequent treatment.

## Materials and Methods

### Random walk models of growth

The model can be used to mimic the growth of an organ or the entire body with fluctuations during the process. The model is a feedback system including a random walker whose steps x are taken from a distribution with time varying parameters. We first define a window of length w during which the parameters of the distribution do not change. The random walk starts with initializing the mean (μ) and variance (σ^2^) of the distribution with values μ(1) and σ^2^(1), respectively, and generating w number of values for x. During the i^th^ window of length w, the walker takes values from a probability distribution written as p(i,x) with mean and variance of μ(i) and σ^2^(i), respectively. Since the growth must approach a finite value, we require that μ(i) gradually decrease approaching 0 with increasing i. Once a long series of x is obtained, the final output of the model is the cumulative running sum of x:

1 $${\rm{g}}({\rm{k}})=\sum _{1}^{{\rm{k}}}\,{{\rm{x}}}_{{\rm{j}}}$$

For convenience, we assume that k is an integer multiple of w, that is, k = nw. The growth process is then represented by the time series of g(k). The value μ(1) can be thought of as the initial level of growth factors (e.g. hormones) present in the system at the beginning of the biological growth process. The window w can be considered as a sensor that measures the average level of growth and the speed of growth is reduced at w intervals. Thus, as μ(i) monotonously decreases in successive windows, the growth slows down. The σ^2^(i) can then be used to generate variabilities representing fluctuations within an individual subject.

To obtain an analytic model, we partition the sum in Eq. 1 into segments of length w.

2 $${\rm{g}}({\rm{k}})=\sum _{1}^{{\rm{w}}}\,{{\rm{x}}}_{{\rm{j}}}+\sum _{{\rm{w}}+1}^{2{\rm{w}}}\,{{\rm{x}}}_{{\rm{j}}}+\ldots +\sum _{({\rm{n}}-1){\rm{w}}+1}^{{\rm{k}}={\rm{nw}}}\,{{\rm{x}}}_{{\rm{j}}}$$

If w is large, we can approximate each sum as the corresponding μ times w:

3 $${\rm{g}}({\rm{k}})={\rm{w}}{\rm{\mu }}(1)+{\rm{w}}{\rm{\mu }}(2)+\ldots +{\rm{w}}{\rm{\mu }}({\rm{n}})$$

which is also the expected value of g(k) in Eq. 2. Furthermore, if the increments in Eq. 2 are not correlated in time, g(k) is a simple sum of independent random variables. Therefore, the variance of g(k) is simply the sum of the variances of each random variables which is constant within the time window of w and hence it can be written as:

4 $${{\rm{\sigma }}}_{{\rm{g}}}^{2}({\rm{k}})={{\rm{w}}{\rm{\sigma }}}^{2}(1)+{{\rm{w}}{\rm{\sigma }}}^{2}(2)+\ldots +{{\rm{w}}{\rm{\sigma }}}^{2}({\rm{n}})$$

For simple choices of μ(i), Eq. 3 can be solved analytically. For example, if μ(i) = δμ(i − 1) with 0 < δ < 1, we can write Eq. 3 as:

5 $${\rm{g}}({\rm{k}})={{\rm{\mu }}}_{0}{\rm{w}}[1+{\rm{\delta }}+{{\rm{\delta }}}^{2}+\ldots +{{\rm{\delta }}}^{{\rm{n}}}]$$

where we introduced μ_0_ = μ(1). This geometric series can be summed up in a closed form

6 $${\rm{g}}({\rm{k}})={\mu }_{0}{\rm{w}}\frac{1-{{\rm{\delta }}}^{{\rm{n}}+1}}{1-{\rm{\delta }}}$$

Finally, for large k = nw, we can replace k with continuous time t and after some manipulations we obtain the following solution:

7 $${\rm{g}}({\rm{t}})=\frac{{\mu }_{0}{\rm{w}}}{1-{\rm{\delta }}}(1-{{\rm{\delta }}e}^{-{\rm{\gamma }}t})$$

where γ = ln(1/δ^1/w^) and hence τ = 1/γ is the time constant of the system. Eq. 7 shows that the time course of the continuous biased and time varying random walk is an exponential saturation. The w can be considered as a time scale of the sensor which smooths the microscopic fluctuations of the growth process. The initial growth rate, μ_0_, only affects the saturation level. Note that the strength of the feedback, δ, enters both the saturation level and the velocity with which the saturation is approached.

Another simple choice for the mean to decrease is a power law form μ(i) = μ_0_i^α^ with α < 0. In this case, Eq. 3 becomes

8 $${\rm{g}}({\rm{k}})={{\rm{\mu }}}_{0}{\rm{w}}[{1}^{{\rm{\alpha }}}+{2}^{{\rm{\alpha }}}+\ldots +{{\rm{n}}}^{{\rm{\alpha }}}]={{\rm{\mu }}}_{0}{\rm{w}}\sum _{1}^{{\rm{n}}}\,{{\rm{i}}}^{{\rm{\alpha }}}$$

Using n = k/w and transitioning to the continuous time domain, Eq. 8 is written as

9 $${\rm{g}}({\rm{t}})={{\rm{w}}{\rm{\mu }}}_{0}{\int }_{1}^{{\rm{t}}/{\rm{w}}}\,{{\rm{\tau }}}^{{\rm{\alpha }}}{\rm{d}}{\rm{\tau }}$$

Eq. 8 is then written in its final form as

10 $${\rm{g}}({\rm{t}})={{\rm{At}}}^{1+{\rm{\alpha }}}+{\rm{B}}$$

which is a power law growth function with A = μ_0_w^−α^/(1 + α) and B = −μ_0_w/(1 + α). However, due to the relation among the 4 parameters given by B = w^1+α^A, only 3 of them are independent. Furthermore, Eq. 10 is similar to the extension of the Faulhaber formula to non-integer powers^32^ but it provides an improved approximation of Eq. 8 with an error <0.6% for a 6 year prediction of g(t) when α = −0.5.

To derive a formula for the variance of the growth curve, we note that Eq. 4, can be written in the same form as Eq. 8 by assuming that σ(i) = sμ(i) where s is a constant. The same arguments can be applied as for the mean and the final result is:

11 $${{\rm{\sigma }}}^{2}({\rm{t}})={{\rm{Ct}}}^{1+2{\rm{\alpha }}}+{\rm{D}}$$

where C = s^2^μ_0_^2^w^−2α^/(1 + 2α) and D = −ws^2^μ_0_^2^/(1 + 2α).

### A computational model of height

To extend the analytical model computationally, we note that the derivative of the power law in Eq. 10 defines the increment distribution of growth. Taking the derivative of the WHO height data and applying a linear regression in the log-log domain, we find that the exponent α is −0.543 with an intercept of 1.037 for the average height of boys whereas these numbers for girls are −0.511 and 0.93, respectively. The WHO data reveal a range of growth rates within the population of both boys and girls; therefore, we also fit the mean ± SD and the mean ± 2SD increment curves, with the power law model. For boys, this gives an α of −0.505 for the mean + 2SD curve and −0.589 for the mean − 2 SD curve whereas for girls, these exponents are −0.483 and −0.546, respectively.

While the fully deterministic power law model captures the mean growth rates, it does not account for the distribution of growth increment amplitudes and waiting times (stasis) at each step of the random walk. We incorporate these stochastic elements into a computational model and demonstrate that in the statistical sense, this model also describes the average height curves in the WHO data^27^ which is then taken as a training set for the model. The overall growth velocity is determined by variations in both the growth increments and the stasis intervals as demonstrated schematically in Fig. 2. The relationship between the amplitude of the random walk steps and length of stasis periods determine the growth vector. Long stasis times and low amplitudes are related to slow growth and via versa. For growth in height, the amplitudes must be non-negative. If the model was applied to describe weight, an occasional loss can also be allowed and hence the amplitudes can take negative values.

Growth increment amplitudes, stasis times and their variations have been described in the literature only in small selected populations of different age groups. In infancy, peak amplitudes of salutatory growth were described to be between 0.46 and 1 cm^14, 24^ and their variations between 10 and 40% while in early school-age these numbers were between 0.2 and 0.42 cm with variations of 10 to 25%^14, 24, 25^. The corresponding stasis times were reported to be between 10 and 17 days with variations of ~10%. However, there is no systematic data on developmental changes of growth increments and mean stasis time, T_stasis_, and their variances σ_ampl_ and σ_stasis_, respectively. These parameters can, however, be estimated by fitting model to the WHO population data. Additionally, due to the strong genetic component of growth^33^, the initial height at birth will be taken to represent genetic predisposition to growth. Thus, a linear regression between α values of the mean and mean ± SD and ±2SD growth curves and the corresponding initial heights at birth provides the genetic background in the model.

To construct a computational random walk model, we use the following assumptions: (*1*) the time variation of the mean of the growth increment distribution is obtained by differentiating the growth function in Eq. 10 with respect to time which is a power law with an exponent α. Note that since the power law analytic model provides a better description of the data (see Fig. 1), only this model is retained for further analysis. (*2*) Since experimental data show a right skewed distribution of height increments^34^, we assume that the increment distribution is lognormal which also guarantees that all increments are positive. (*3*) We assume that the distribution of stasis times is also lognormal with a mean T_stasis_ and variance σ_stasis_. Assigning specific values for these parameters and knowing the height at birth that determines α through the regression as described above, we can simulate individual growth curves. Thus, using 200 random walks for a single subject, we compute the expected growth curve of the subject by ensemble averaging. Since the population distribution in the WHO data set for heights is normal^27^, we mimic the growth of 100 infants with a normal distribution of initial heights at birth (mean height is 49.88 cm and SD is 2.1 cm). Summing up all these growth curves, we determine the least square error between the simulated population and the mean and ±2SD height trajectories of the WHO data which allows us to optimize these parameters to best describe the WHO population. Once the model is set up, we introduce an additional feature of the model, an adaptive correction mechanism that accounts for environmental influences as described in the Results section. Finally, the model is used to predict height at 6 years of age in an independent population data set.

### Experimental data

In a prospective birth cohort (BILD)^29^ of preschool children (40% boys), height, weight, BMI was measured at the age of 5 weeks and at the age of 6 years using standard measurement techniques (Table 1). The cantonal ethics committee of the University of Basel and the Children’s Hospital approved the study. All methods were performed in accordance with the relevant guidelines and regulations and written informed consent was obtained from all parents. 40% of the children were boys.
